# Organisational Culture in Residential Aged Care Facilities: A Cross-Sectional Observational Study

**DOI:** 10.1371/journal.pone.0058002

**Published:** 2013-03-07

**Authors:** Christopher Etherton-Beer, Lorraine Venturato, Barbara Horner

**Affiliations:** 1 Western Australian Centre for Health and Ageing, The University of Western Australia, Crawley, Western Australia, Australia; 2 School of Nursing and Midwifery, Griffith University, Brisbane, Queensland, Australia; 3 Research Team, RSL Care, Brisbane, Queensland, Australia; 4 Centre for Research on Ageing, Curtin University, Perth, Western Australia, Australia; University of Valencia, Spain

## Abstract

**Background:**

Organisational culture is increasingly recognised as important for provision of high-quality long-term care. We undertook this study to measure organisational culture in residential aged care facilities in two Australian states.

**Methodology/Principal Findings:**

Cross-sectional observational study in 21 residential aged care facilities in Western Australia (n = 14) and Queensland (n = 7), Australia. Staff and next-of-kin of residents participated. Measurement comprised surveys of facility staff and residents' next-of-kin, and structured observation of indicators of care quality. Staff tended to rate organisational culture positively. Some qualitative feedback from staff emphasised negative perceptions of communication, leadership and teamwork. Staffing levels were perceived as a dominant challenge, threatening care quality. Direct observation revealed variability within and between facilities but suggested that most facilities (n = 12) were in the typical range, or were quality facilities (n = 8).

**Conclusion:**

There was scope to strengthen organisational culture in participating aged care facilities.

## Introduction

Although older people often enjoy productive and healthy longer lives, population ageing presents many challenges. Residential care is an important component of service delivery for older people with complex health problems, particularly severe dementia [1]. Costs, staff shortages, staff turnover and communication problems are cited as barriers to sustainable improvements in the care of people living in residential care facilities [2]. Although educational interventions in residential care may be evaluated positively by participants [3], we found evidence that impact on residents may be restricted by limited staff participation [4]. Focusing on sustainable culture change may offer an alternative method of quality improvement in residential care settings.

Organizational culture refers to the psychology, attitudes, experiences, beliefs and values (personal and cultural) of an organization [5]. We found that teamwork, communication and leadership were consistently recognised as key elements of organisational culture which potentially influence staff and resident outcomes in care facilities [2]. Other empirical evidence supports these findings, showing that management behaviour (such as the extent of open communication patterns and relationship-oriented leadership behaviour) is associated with resident outcomes [6]. Systematic review of the evidence regarding leadership indicates that leadership styles focused on people and relationships are associated with more positive outcomes than leadership styles focused on tasks [7].

Organisational culture is increasingly recognised as important for the provision of high quality long-term care [8], [9]. However, there are few data regarding the prevailing organisational culture in Australian residential care facilities. Thus a knowledge gap persists regarding organisational culture, and the potential impact of quality improvement initiatives targeting the organisational culture, in Australian long-term (residential) aged care facilities. We undertook the present study to measure organisational culture in residential aged care facilities in two Australian states.

## Methods

### Ethics

This research was approved by the Human Research Ethics Committees of the Universities of Western Australia, Curtin University and Griffith University. Representatives of all participating facilities provided written consent.

### Study design & setting

A cross sectional observational study conducted in a convenience sample of 21 RCFs in Western Australia (n = 14) and Queensland (n = 7). The sample included 2 high care (“nursing home”) only facilities, 8 low care (“hostel”) only facilities and 11 facilities providing both high and low level care. The median number of beds in participating facilities was 70 (IQR 60–94).

### Participants

In Western Australia, 20 control facilities from a previously conducted study [10] were invited to participate. These facilities were owned and operated by diverse providers. Telephone contact was initially made with facility managers and a brief overview of the study presented. All managers agreed to a follow up email being sent providing more detailed written information regarding the study.

In Queensland, seven facilities were invited to participate. Facilities were owned and operated by a single not-for-profit aged care provider and recruited in the context of an existing, long-standing research partnership with one author (LV). Invited facilities were accessible (located in South-East Queensland) and not engaged in other research projects at that time. Contact with facilities was initially made through Area Managers, who were provided with information on the study. This was followed up by email and telephone calls to facility managers to provide more information on the study. All facility managers agreed to participate.

After facilities had consented to participate, an on-site meeting was held between facility managers and research staff. An information session regarding the study was then held at each facility (staff, family members and residents were welcome to attend these) to profile the study, encourage participation, answer any queries raised and distribute flyers and posters regarding the study. All staff working in participating facilities (unless temporary, agency or short term contract staff), as well as the ‘next-of-kin’ of all residents were eligible to participate in the study.

### Data collection

Baseline data included surveys of all staff and family members (‘next-of-kin’) at each facility and independent observation using a structured observation tool. Data were collected in August and September 2010.

The staff survey comprised the Nursing Home Adaptation of the Shortell Organisation and Management Survey, a valid and reliable tool [11] which has been used extensively in the United States [12]. The Nursing Home adaptation comprises a 15 item Relationships and Communication scale and 11 Item Teamwork and Leadership scale. The introduction to the survey was amended slightly for the Australian context with permission of Dr Jill Scott-Caziewell (personal communication). The introduction to these questions was ‘*This section refers specifically to ‘nurses’ but the questions are relevant to everyone. For example you may think of the ‘nursing leadership’ as people like your Facility Manager, Director, Assistant Director or Charge Nurse. Similarly ‘nurses’ refers to carers, enrolled nurses and registered nurse.*’ Staff surveys also included the Engagement/Empowerment and Team communication factor items of the Healthcare Team Vitality Instrument. This is a valid [13] instrument which was developed to provide a short tool to assess aspects of teamwork and collaboration. The actual survey items are provided in Table 1.

**Table 1:** Staff responses to individual survey items [n (%)].

	Strongly Disagree n(%)	Dis-agree n(%)	Unsure n(%)	Agree n(%)	Strongly Agree n(%)
*Shortell – Relationships and Communication*					
I look forward to working with our staff each day.		16 (5)	27 (8)	221 (63)	89 (25)
It is easy for me to talk openly with our staff.	2 (1)	19 (5)	42 (12)	209 (60)	79 (23)
There is good communication between staff across shifts	10 (3)	61 (17)	81 (23)	167 (47)	33 (9)
I feel that the information that I get is accurate		32 (9)	60 (17)	228 (65)	31 (9)
I find it enjoyable to talk to other staff	1 (0)	7 (2)	29 (8)	233 (66)	82 (23)
Staff members are well informed about what I happening during other shifts	6 (2)	75 (22)	83 (24)	158 (45)	26 (7)
Information passed between staff is accurate	4 (1)	42 (12)	104 (30)	182 (53)	14 (4)
It is easy to ask for advice from other staff	5 (1)	20 (6)	36 (10)	231 (66)	56 (16)
When a resident's condition changes, I get the right information quickly.	10 (3)	52 (15)	55 (16)	182 (52)	48 (14)
I take pride in being a part of this team.		10 (3)	31 (9)	184 (52)	126 (36)
The staff has a good understanding of goals for each resident.	1 (0)	35 (10)	76 (22)	186 (53)	50 (14)
There are no delays in relaying information aboutthe care of the residents.	6 (2)	48 (14)	84 (24)	174 (50)	35 (10)
I identify with the goals of this nursing home.	3 (1)	8 (2)	32 (9)	207 (59)	98 (28)
I feel I am a part of this team.	3 (1)	20 (6)	28 (8)	182 (52)	117 (33)
The staff has a good understanding of the resident care plan.	2 (1)	33 (9)	59 (17)	196 (56)	60 (17)
*Shortell – Teamwork and Leadership.*					
Nursing Leadership provides strong clinical guidance and advice to the nurses.	7 (2)	19 (6)	56 (17)	197 (58)	59 (17)
Nursing leadership is sensitive to the needs of staff	10 (3)	33 (10)	68 (20)	167 (50)	54 (16)
Nursing leadership is clear about what they expect from staff	9 (3)	24 (7)	55 (17)	182 (55)	62 (19)
Nursing leadership encourages nurses to take initiative	8 (2)	26 (8)	66 (20)	182 (55)	50 (15)
Nursing leadership asks us what we think	18 (5)	37 (11)	79 (24)	156 (47)	44 (13)
Nurses are certain where they stand with the nursing leadership	9 (3)	27 (8)	81 (24)	179 (54)	35 (11)
The nursing leadership is in touch with staff views and concerns	19 (6)	44 (13)	77 (23)	156 (47)	38 (11)
Nursing leadership makes decisions with input from the staff	12 (4)	37 (11)	84 (25)	153 (46)	45 (14)
Nursing leadership gives staff chances to grow	16 (5)	29 (9)	68 (20)	167 (50)	53 (16)
Other nursing homes seem to have a high opinion of us	5 (1)	16 (5)	140 (42)	120 (36)	55 (16)
Working as a team with other departments makes our work easier.	4 (1)	15 (4)	48 (14)	197 (59)	72 (21)
*HTVI – Engagement/Empowerment*					
If I have an idea about how to make things better on this unit, the manager and other staff are willing to try it.	4 (1)	41 (12)	76 (22)	182 (53)	40 (12)
My ideas really seem to count on this unit.	12 (4)	41 (12)	93 (28)	163 (48)	29 (9)
Care team members on this unit feel free to question the decisions or actions of those with more authority	20 (6)	41 (12)	86 (25)	156 (46)	37 (11)
*HTVI – Team Communication*					
I can discuss challenging issues with care team members on this unit.	5 (1)	23 (7)	38 (11)	207 (61)	61 (19)
I speak up if I have a patient safety concern.	1 (0)	2 (1)	9 (3)	173 (51)	153 (45)

The next-of-kin survey comprised the F-involve scale [14], a valid and reliable measure of families' perceived involvement in care. With permission of Dr Colin Reid (personal communication), the F-involve scale was modified slightly (adding “not applicable” to the last three items, which were dementia specific, and allowing respondents to select “unsure” i.e. a five point Likert scale).

As this was the first use of these tools in Australia that we were aware of we assessed whether ‘the survey captured my opinions regarding leadership and communication in our organisation’ and whether ‘the survey captured my opinions regarding my involvement in my family member's care’ (using 5 point Likert scales). In both the F-Involve and modified Shortell surveys, we also assessed: time taken to complete the survey; whether the ‘the survey questions are easy to understand’; and space was provided for free text ‘comments you have regarding the survey questions or format’.

Surveys were distributed to all staff (n = 1891) and next-of-kin (n = 1501) by their facility managers. However 220 of the next-of-kin surveys included an error (‘strongly disagree’ was printed instead of ‘agree’). Thus, 1281 correct next-of kin surveys were distributed to facilities. Distribution of staff surveys was most often by attaching surveys to staff payslips. Facility mailing lists were used to send the survey to the recorded next-of-kin. Survey completion was encouraged by postcards and an incentive was provided (in the form of a draw for a voucher). RACF staff returned 356 surveys (response rate 19%). 331 correctly printed next-of-kin surveys were returned (26%). 66 incorrectly printed next-of-kin surveys were returned (30%) and discarded.

Facilities were observed using the Observable Indicators of Nursing Home Care Quality Instrument (OIQ) [15]. This 30 item instrument requires walk through tours of each facility being surveyed for 20 to 30 minutes during usual visiting hours preferably near a mealtime. Two observers, who were authors or research staff (see acknowledgements), first met with the facility manager and then conducted the walk through observations. Field notes were made during the walk through regarding observers' impressions, general ambience of facility, the level of engagement of staff, activities of residents (both organised and informal), and physical attributes of the facility. The two observers then independently rated the OIQ, before meeting to discuss their ratings question by question and together complete a third consensus rating. Each of the 30 items in rated on a 5 point scale (1–5). Total scores are then categorised in three groups. A total score≥128 suggests a quality nursing home. Scores ≤103 suggest a nursing home with quality issues. Scores between these numbers are typical of most nursing homes.

### Data handling and analysis

Quantitative survey data were read by optical scanning. Quantitative data were handled in PASW Statistics 18 (IBM Corporation, New York, USA). Staff survey scale scores were calculated by summing the individual items and dividing by the number of non-missing items. Total F-involve scores were calculated by summation (not applicable, unsure and missing data scoring zero), and then scaled (*20/number of non-zero responses) to produce scores that could be compared to the original scale. Internal reliability was calculated (Cronbach's alpha). We also inspected correlations between scales.

Observational data sets from 21 sites were randomly allocated amongst the WA and Queensland research teams and coded independently by two study staff. Research staff independently read and analysed data sets from each facility for key words and phrases, then independently analysed key words and phrases across selected facilities to reveal common themes. Staff then met with the authors from each State to review coding and common themes and to reach consensus. Themes agreed from each State where then discussed, combined and consensus reached across both States regarding the summary themes and illustrative uncoded data.

Free text responses to the surveys were transcribed and randomly allocated for independent coding by two members of the study staff. The analysts then met to review each other's coding, reach consensus and create a thematic summary. All staff members and authors then reviewed and agreed the final summary of themes (and selections of illustrative uncoded data) from the survey data.

## Results

### Survey of facility staff

Respondents were largely personal carers or nursing staff (152 [43%] carers, 40 [11%] registered nurses and 17 [5%] enrolled nurses). Allied health and therapy assistant staff (n = 26; 8%), support staff (cleaning, catering, laundry and maintenance n = 42; 12%), administrative and management (n = 53; 15%) and other staff (n = 20; 6%) also responded. Few staff were young (19 [5%] ≤25 years, 33 [9%] 26–35 years, 69 [19%] 36–45 years, 112 [31%] were aged 46–55 years, 74 [21%] 56–65 years, 10 [3%] ≥65 years, 39 [11%] missing). The median length of service was 2.8 (IQR 1.3, 5.8) years.

Staff reported positive relationships and communication at their facility (Table 1; median Shortell scale score 3.8, IQR 3.5, 4.2). Team work and leadership were also rated positively (median scale score 3.7, IQR 3.3, 4.1). The median total Shortell scale score was 3.8 (IQR 3.5, 4.1). Reported engagement/empowerment (median HTVI scale score 3.7; IQR 3.0–4.0) and communication (median 4.0, IQR 4.0, 4.5) were also positively assessed using the Healthcare Team Vitality Index.

Some qualitative feedback from staff emphasised negative perceptions of aspects of communication, leadership and teamwork. Teamwork was sought and valued, but sometimes perceived to be lacking. Communication was perceived as challenging at times, with some attempts at communication ineffective:

*‘Staff meetings are intimidating and methods of communication “tell us what you think” forms are ridiculed’*

Staff levels were perceived as a dominant challenge, threatening care quality:

*‘As usual, the main complaint appears (in my opinion) to be poor staffing in patient/carer ratios. Although staff are concerned and caring, pressure of work results in cut back to care time available’*

The importance of leaders establishing and maintaining the culture of an organisation was emphasised. Leadership was perceived to have concrete influences on organisations in areas such as staff retention.

*‘This facility is poorly managed in every area….32 staff members have resigned in the last 6 weeks’*

Despite these difficulties and challenges, resilient personal values were emphasised:

*‘I am proud to work at* <organisation name>, *dedication, honesty, trust’*

### Survey of family involvement in care

Responses were received from 331 family members, who reported that their relative had lived in the facility 1.7 (0.8, 3.1) years. (Table 2) Total F-involve scores were 46.1±11.9. The scaled (i.e. corrected for missing items, and answers of “not applicable” or “ unsure”) scores were 55.3±11.4.

**Table 2:** F-involve results.

	Strongly Disagree	Somewhat Disagree	Somewhat Agree	Strongly Agree	Does not apply
Staff have created opportunities for me to meaningfully participate in my family member's day.	5 (2%)	48 (15%)	37 (11%)	177 (55%)	N/A
I have been asked about my family member's personal history	13 (4%)	43 (13%)	25 (8%)	168 (52%)	N/A
I have been asked about my family member's preferences and values	12 (4%)	50 (15%)	37 (11%)	158 (48%)	N/A
I am able to dine with my family member if I want to	4 (1%)	16 (5%)	45 (14%)	162 (50%)	N/A
I have been asked to bring in pictures, letters, and other personal items to teach staff about my family member	33 (10%)	119 (37%)	34 (11%)	92 (29%)	N/A
I feel like I am involved in decision-making about my family member's care when he or she cannot make decisions for themselves	12 (4%)	38 (12%)	24 (7%)	181 (56%)	N/A
The facility has a support group	15 (5%)	34 (10%)	163 (50%)	85 (26%)	N/A
I was introduced to the different staff members at the facility when my family member was admitted	14 (4%)	79 (24%)	13 (4%)	159 (49%)	N/A
Staff explained to me the rules and procedures at the facility upon admission	8 (2%)	42 (13%)	28 (9%)	176 (54%)	N/A
Administrators have asked my opinions about the quality of care provided at this facility	28 (9%)	99 (30%)	32 (10%)	122 (37%)	N/A
The facility holds family information meetings	15 (5%)	54 (17%)	79 (24%)	130 (40%)	N/A
I feel like my family member has been well cared for	4 (1%)	13 (4%)	22 (7%)	165 (50%)	N/A
I trust the staff members at this facility	4 (1%)	10 (3%)	32 (10%)	179 (55%)	N/A
I am informed about changes in my family member's care plan	14 (4%)	67 (21%)	33 (10%)	150 (47%)	N/A
Staff have helped me to plan for the death of my family member	31 (10%)	142 (47%)	57 (19%)	61 (20%)	N/A
Staff have helped me to plan for the handling of my family member's estate upon his or her death	51 (17%)	176 (60%)	47 (16%)	16 (5%)	N/A
I feel comfortable phoning staff members and talking to them about how my family member is doing	4 (1%)	23 (7%)	22 (7%)	190 (58%)	N/A
Staff have helped me understand the difficult behaviours that my family member sometimes has	13 (4%)	52 (16%)	26 (8%)	137 (42%)	34 (10%)
Staff have taught me how to communicate with my family member as the disease has progressed	24 (7%)	84 (26%)	38 (12%)	54 (17%)	12 (4%)
Staff have helped me to understand how dementia affects my family member	23 (7%)	81 (25%)	23 (7%)	76 (23%)	18 (6%)

Data are n (% of total). Rows that sum to <100% indicate missing data. N/A = not applicable.

Respondents were concerned that facilities did not necessarily act on information provided, and suggested solutions to facilitate communication, such as providing family members of new residents with a list of facility contacts.

*‘As my mother has been in aged care for over 5 years, at some point in that time some of the information has been supplied. Is it just filed away?’*

Like facility staff, family respondents focused on staff mix, turnover and workloads as major challenges impacting communication and care.

*‘a lot of staff changes at <facility name> which has confused me let alone my mother who is confused some of the time’*

*‘I find staff are too busy to talk about family members and that I am a nuisance.’*

Family respondents also identified potential to strengthen teamwork.

*‘a major problem also appears to be barriers to effective communication and action between different staff categories e.g. nursing, carers, cleaning, kitchen, with each seeming to operate within their own cells most of the time rather than more effectively as a team’*

Respondents did emphasise quality aspects of care they observed.

*‘The critical aspect of care from my perspective is the quality of affection, respect shown to my mother. It's not so evident in the formal structures but in the minor day-to-day attentions she receives’*

Being welcome to visit the facility and participate in the community there was highly valued.

*‘Something I appreciate very much is that I feel welcome arriving there at any time and being able to take part in whatever is going on at the time, and, that all the staff are really friendly and willing to assist with any enquiry’*

### Observation at facilities

The mean score for the OIQ in the 21 facilities was 124.5±8.7. One facility scored below 104 (suggesting quality issues), and 8 scored above 127 (suggesting a quality nursing home). The remaining 12 scored in the typical range.

Field notes emphasised variability within and between facilities.

*‘Resident staff interaction was really variable across the facility. Where staff responded well to residents their engagement and communication was caring and appropriate…… while in High Care there was almost no engagement with residents. Staff fed in silence and did not engage with the resident at all’*

The physical environment and design was a frequent theme, perceived to have some potential to influence staff work and interactions, as well as resident care. However there was not necessarily correlation between the physical environment and the interactions observed between staff and other staff or residents, or the wellbeing of residents. For example, physical spaces (such as gardens and outdoor areas) were not necessarily accessible or used by residents. Instead, relationships and community were emphasised as having the potential to impact resident wellbeing regardless of the physical environment:

*‘Physically lovely facility but quite unfriendly, no obvious evidence of resident engagement in facility’*

Communal dining experiences were recognised as often being indicative of the broader impression of community at the facility. Management and leadership was also an important theme in the observers’ field notes, including the influence of visible leadership on the facility. Generally, there was thought to be correlation between the leadership style and the interactions observed in the facility.

*‘Calm, serene manager. Calm, serene facility’*

### Acceptability of measurement tools in this Australian context

Facility staff reported that the survey took a median of 9 (IQR 5, 10; minimum 1, maximum 30) minutes. (Table 3) Internal reliability of the scales was generally acceptable: Cronbach's alpha was 0.96 for the Shortell Scale, 0.85 for the HTVI-EE, 0.524 for the HTVI-TC and 0.825 for the HTVI items. Shortell scales scores correlated significantly with HTVI-EE (r = 0.89, p<0.001) and HTVI-Comm (r = 0.72, p<0.001). Staff rated communication was weakly positively associated with the observed care quality (r^2^ = 0.097 for the Shortell total scales score; 0.056 for the HTVI-EE and 0.028 for the HTVI-Communication)

**Table 3:** Feedback on survey tools.

	Strongly Disagree	Disagree	Unsure	Agree	Strongly Agree
*Communication and Leadership Subscales*					
The survey questions are easy to understand	1 (0%)	10 (3%)	8 (2%)	232 (66%)	101 (29%)
The survey captured my opinions regarding leadership and communication in our organisation.	2 (1%)	19 (6%)	40 (12%)	217 (63%)	66 (19%)
*F-involve survey*					
The survey questions are easy to understand	2 (1%)	6 (2%)	3 (1%)	236 (71%)	80 (24%)
The survey captured my opinions regarding my involvement in my family member's care	4 (1%)	19 (6%)	41 (12%)	213 (64%)	43 (13%)

Respondents perceived greater relevance of the survey in high level care facilities. Similarly there was concern that the questions may have limited relevant to some groups of staff (such as non-care staff). Some respondents felt that the questions did not allow them to indicate the variability present in their facility.

Family respondents reported that the F-involve survey took a median of 5.5 minutes (IQR 5, 10; minimum 1, maximum 60). Some family respondents contended that “not applicable” should be provided for all questions, feeling that the survey remained too dementia specific. Several respondents felt questions could be answered with yes/no responses.

Research staff using the OIQ questioned the usefulness of the tool. They felt that the tool did not facilitate adequate assessment of the physical environment or communication. Measures of physical spaces (such as gardens and outdoor areas) were difficult to rate and differentiate and the relationship to level of care and resident engagement was difficult to determine. There was great variation in design as well as environment across facilities and this was difficult to assess. The OIQ was not perceived effective in measuring leadership, even though there appeared to be a correlation between the leadership style and the interactions observed in the facility. The influence of visible leadership on staff and residents was described as ‘*palpable*’ but impossible to score. Research staff felt that the specific staff rostered at the time of observation may influence results, as did the unit type observed at mealtime (eg. high care or low care unit). Applicability of some items in the tool in low level care environments was particularly uncertain.

## Discussion

### Main findings and interpretation

We found evidence that several aspects of organisational culture are rated positively in Australian long-term aged care facilities by both staff working in facilities, and the next-of-kin of residents. However, there was variability within individual participating facilities, and within the data collected, which included both positive and negative perceptions of aspects of organisational culture.

The qualitative data revealed substantial overlap between issues cited as important by both staff and family participants, such as communication, leadership and team work.

Leadership was consistently perceived to be important and seemed to impact on concrete outcomes such as staff retention. Existing organisational culture appeared to be expressed in teamwork and care delivery. Similarly, observation within facilities also suggested that organisational culture could impact resident wellbeing.

### Implications

As far as we are aware this is the first systematic study of organisational culture in Australian long term aged care facilities. Application of these measurement tools is novel in an Australian context. The present study suggests that these tools have broad acceptability in the Australian context. The OIQ tool was perceived to have some limitations in relation to the areas of particular interest in the present study (such as leadership). These novel data indicate scope to specifically target improvements in organisational culture (seeking to improve teamwork, communication and leadership) in Australian residential aged care facilities.

### Strengths and limitations

This study was comprehensive, including triangulation of data, investigators and methods. The breadth of participants, including both staff and families, increases the reliability of results. Including residents in future data collections would further enhance the opportunities available to triangulate data sources. Gathering data from several sites and across states enhances generalisability of results to some extent. However, response rates for staff surveys were relatively low (19%) limiting generalizability. Furthermore, invitations to participate in the study were not random or universal, potentially introducing a selection bias and thus limiting external generalizability. We also did not collect administrative data from participating facilities (such as staff ratios and turnover) that may strengthen future studies. Although participants expressed some concerns about the tools utilised, these were relatively minor. The collection of feedback regarding the tools used, and free text data in addition to the Likert scale responses, enriched the data available and is a strength of the present study.

### Results in context of other studies

The term culture change is usually used to indicate a fundamental reform process targeting attitudes and behaviour. Promoting resident choice has been an important component of many cultural change interventions in residential care settings [9], [16]. Others promote a change of philosophy with a focus on normal activities in a home like environment [16]. However there are few other data regarding fundamentals staff interactions (teamwork, communication and leadership).

Our data are consistent with those suggesting that leadership can be harnessed to transform organisational function [17]. However, most work regarding organizational strategy has been done in the acute care sector and little is known regarding how residential care facilities identify and implement strategies to improve leadership. Similarly, our data are consistent with the empirical studies that have targeted improved communication. For example, a study targeting improved cooperation between staff and families of residents in nursing home dementia programs found positive outcomes for all groups [18]. The findings of the present study are also consistent with studies from other countries suggesting that improved communication and leadership are required for nursing homes to continue to develop as organizations pursuing quality improvement [12].

### Key points

- Several aspects of organisational culture were rated positively in participating long-term aged care facilities.
- There was variability between and within facilities.
- Existing organisational culture appeared to be expressed in care delivery and teamwork.
- Teamwork, communication and leadership are potential targets of specific interventions to enhance organisational culture in Australian residential aged care facilities.
